# C-Fos expression in epileptogenic areas of nephropathic rats undergoing star fruit poisoning ^1^

**DOI:** 10.1590/s0102-865020200070000005

**Published:** 2020-08-10

**Authors:** Renato Figueiredo Santana, Layla Alves Rodrigues da Silva, Eduardo Achar, Gerson Ballester, Marcelo Augusto Fontenelle Ribeiro, Sandra Regina Mota Ortiz

**Affiliations:** I Fellow PhD degree, Postgraduate Program in Health Science, Instituto de Assistência Médica ao Servidor Público Estadual (IAMSPE), and Associate Professor, Universidade Cidade de São Paulo (UNICID) and Universidade São Judas Tadeo (USJT), Sao Paulo-SP, Brazil. Scientific, intellectual, conception and design of the study; acquisition, analysis and interpretation of data; histopathological examinations; statistics analysis; manuscript preparation and writing, final approval.; Universidade São Judas Tadeo, Sao Paulo, SP, Brazil; II Master, Health Sciences, IAMSPE, Sao Paulo-SP, Brazil. Scientific and intellectual content of the study; acquisition, analysis and interpretation of data; histopathological examinations; technical procedures.; III PhD, Associate Professor, UNICID and Universidade de São Caetano do Sul (USCS), Sao Paulo-SP, Brazil. Conception and design of the study, technical procedures.; Universidade de São Caetano do Sul, Sao Paulo, SP, Brazil; IV PhD, Associate Professor, UNICID, Sao Paulo-SP, Brazil. Conception and design of the study, technical procedures.; V Full Professor, Department of Surgery, Pontifícia Universidade Católica de São Paulo (PUC-SP), Sorocaba-SP. Associate Professor of Surgery, Postgraduate Program in Health Science, IAMSPE, Sao Paulo-SP, Brazil. Scientific and intellectual content of the study, manuscript preparation and writing, critical revision, final approval.; Postgraduate Program in Health Science, IAMSPE, Sao Paulo, SP, Brazil; VI PhD, Full Professor, USJT, and Assistant Professor, Postgraduate Program in Health Science, IAMSPE, Sao Paulo-SP, Brazil. Scientific, intellectual, conception and design of the study; manuscript preparation and writing; critical revision; final approval.

**Keywords:** Averrhoa, Seizures, Kidney Diseases, Neurotoxicity Syndromes, Rats

## Abstract

**Purpose:**

Studies have demonstrated that star fruit consumption by nephropathic patients triggers severe neurotoxic effects that can lead to convulsions or even death. Brain areas likely susceptible to star fruit poisoning have not been investigated. The objective of the present study was to map possible epileptogenic areas susceptible to star fruit intoxication in nephropathic rats.

**Methods:**

The study analyzed 25 rats (5 groups). Rats in the experimental group underwent bilateral ureteral obstruction surgery and orogastric gavages with star fruit juice. An electroencephalogram was used to confirm convulsive seizures. Urea and creatinine levels were used to confirm the uremia model. Immunohistochemical analysis was used to map cells with c-Fos protein (c-Fos+ cells) to identify brain areas with increased neuronal activity. Control groups included non-nephropathic and nephropathic rats that did not receive star fruit.

**Results:**

A statistically significant increase (p<0.01) in c-Fos+ cells was noted in nephropathic animals receiving star fruit juice compared to control groups, in brain areas commonly related to epileptogenic neural circuits including the hippocampus, amygdala, rhinal cortex, anterior cingulate area, piriform area, and medial dorsal thalamus.

**Conclusion:**

These data corroborate the neurotoxic capacity of star fruit in nephropathic patients.

## Introduction

Star fruit ( *Averrhoa carambola* ) is a species of fruit tree that belongs to the Oxalidaceae family. Having originated in Asia, its fruit is popular in tropical and subtropical countries. Star fruit can be consumed as a fruit in its natural form or as a juice^1,2^. In the 1980s, the first report of neurotoxic effects originated from administration of constant intracerebroventricular injections of star fruit juice in healthy rats, inducing seizures and suggesting the existence of a neurotoxic compound in the fruit^3^. In the following years, several case reports were described in uremic patients who had ingested star fruit or its juice^3- 9^ .

Star fruit poisoning has been classified as mild, moderate, or severe according to the clinical signs presented by the patient, progressing to vomiting, mental confusion, psychomotor agitation, seizures, hypotension, coma, and death. The mortality rate was as high as 75%^3,4, 7 - 9^ in patients who developed an epileptic status. Fang *et al* .^9^ showed that nephrectomized rats that received star fruit or oxalate extract developed myoclonic or tonic-clonic seizures.

Historically, two mechanisms are involved in the pathophysiology of seizure attacks possibly caused by the neurotoxic effects of star fruit: 1) the existence of a specific star fruit toxin (caramboxin) inhibiting GABAergic receptors, and 2) the role of oxalic acid (oxalate) in possibly inducing convulsive events^2, 9 - 12^ .

Upon examining case reports of nephropathic patients who developed neurological symptoms after ingesting star fruit, the presence of a seizure attack appears to be a factor of poor prognosis for mortality. Since no studies have so far identified the neural sites involved in seizures in experimental models under these conditions, the objective of this study was to identify brain areas possibly involved in convulsions resulting from the neurotoxic effect triggered by carambola juice intake in nephropathic rats.

## Methods

All experiments were conducted in compliance with the rules and precepts of the Brazilian College of Animal Experimentation (COBEA) to avoid and minimize animal suffering as much as possible. This study was approved by the Committee on Animal Research and Ethics (CARE - UNICID) under n. 001/2015 on August 19, 2015.

A total of 25 male Wistar rats were used. The animals were kept in the *vivarium* of UNICID under a 12/12 light-dark cycle, with controlled temperature, and with water and food *ad libitum* .

### Group design

All animals were habituated to initial conditions. Day 1 conditions were the same for all animals. The animals were divided into different groups, as follows:

1. **White control (WHI):** animals under no procedure - white (WHI). This group did not receive any kind of experimental intervention during their lifetime.
2. **Water gavage control (WGV).** This group underwent water gavage procedures on days 9 and 10.
3. **Star fruit gavage control (SGV).** This group underwent star fruit gavage procedures on days 9 and 10.
4. **Nephropathic gavage control with water (NPW).** On day 8, this group had ureters mechanically blocked and sectioned using a surgical procedure (bilateral ureteral obstruction surgery, BUO). During days 9 and 10 these animals received water gavages.
5. **Experimental group: nephropathic gavage with star fruit (NPS).** On day 1, this group underwent electrode implantation for recording the electroencephalographic pattern. On day 8, the group underwent BUO. During days 9 and 10, these animals received star fruit juice gavages. On day 10, an electroencephalogram (EEG) was also performed.

On day 10, all groups underwent a cardiac perfusion and the brain was removed for subsequent microtomy and immunohistochemical procedures.

Plasma urea and creatinine levels were measured to experimentally validate that the NPW and NPS groups were in a condition similar to nephropathy. Likewise, an EEG was recorded to experimentally validate that the rats had a seizure. The data obtained from these procedures served as a positive control of nephropathy and seizure.

### EEG (NNPS - implant on day 1 and record on day 10)

In order to confirm the presence of seizures, the following brain areas were selected for bilateral implantation of electrodes (day 1) and EEC (day 10): hippocampus, CA1 (AP: -4.6; LAT: 2.5; PROF: 3.0), and primary motor cortex (AP: -2.0; LAT: 1.5; PROF: 2.0), using the Swanson’s atlas as reference, 1998^13^ . These structures were chosen due to comprehensive existing description of the electroencephalographic procedures and standards for these regions (14,15). The electrodes were implanted on experimental day 1 and recordings were always performed during day 10 after electrode implantation using a Nihon Kohden 4412P Neurofax device. This period was selected to reduce the inflammation resulting from electrode implantation, which could influence the EEG signal acquisition and the expression of c-Fos protein. All procedures were performed as described by Fonoff *et al* .^14^ and Timo-Iaria *et al* .^15^ .

### BUO (NPW and NPS, day 8)

Bilateral ureteral obstruction surgery (BUO) was performed on day 8 in the NPW and NPS groups. The animals were anesthetized, the abdominal cavity was exposed, and the ureteral lumen was obstructed with two sutures in its middle third and one section between these two sutures, as described by Achar *et al* .^16^ .

### Gavages and star fruit juice administration (WGV, SGV, NPW, NPS, days 9 and 10)

The gavages were performed using a metallic orogastric cannula on days 9 and 10. The WGV and NPW groups received water gavages. The SGV and NPS groups received star fruit juice gavages. The star fruit juice was produced by mixing the fruit in a blender for a few minutes without addition of any other components. The final product was filtered using a three-micron filter paper. Each animal received 1 mL of liquid (water or star fruit) every three hours, maintaining an unrestricted supply of water. The gavages were performed at the following times: 7:30 AM, 10:30 AM, 1:30 PM, and 4:30 PM on the first day; and 7:30 AM, 10:30 AM, and 1:30 PM on the second day.

### Perfusion and microtomy (days 10 and 11, respectively - all groups)

For immunohistochemical studies, the animals were anesthetized with dissociative general anesthesia using ketamine hydrochloride (100 mg/ml) at a dose of 70 mg/kg of body weight, along with xylazine hydrochloride (2 g/100 ml) at a dose of 10 mg/kg of body weight via intracardiac perfusion with buffered saline (pH 7.4), subsequently using a fixing paraformaldehyde solution (PFA, 4%) at 4°C. During the initial perfusion, approximately 3 mL of blood were collected from the left ventricle to measure urea and creatinine. After the perfusion, a craniotomy was performed, and the brain was maintained for about four hours in 4% PFA (4ºC). The brain was then kept for about 24 hours in a solution with 30% sucrose in phosphate buffer pH 7.4 (PB), in which it remained until it was sectioned at 40 µm thickness using a freezing microtome. The sections were kept in 0.1 M phosphate buffer, pH 7.4 (0.1 M PB) at 4°C or in an anti-freeze solution.

### Dosage of urea and creatinine (all groups - after perfusion)

Commercial kits (Ureia CE and Creatinina K - Labtest Diagnóstica) were used to measure urea and creatinine levels. The results of these measurements were used to calculate the amount present in the sample in mg/dL. The working and dosing reagents were prepared strictly following the procedures indicated by the manufacturer.

### Immunohistochemical procedures (all groups starting on day 10)

The brain sections were washed in PB buffer and incubated in normal 2% donkey serum (Jackson ImmunoResearch) and primary Rb x c-Fos antibody (Calbiochem^®^) at a dilution of 1:10,000 for 48 hours at 4°C. Dilutions were always prepared with 0.3% Triton-X-100 in 0.1 M buffer. The sections were washed again and incubated for one hour in a biotinylated secondary antibody Dk x Rb solution (Jackson ImmunoResearch) at 1:250 dilution in 0.1 M buffer. After washing again, the tissue sections were incubated for one hour in a solution containing the avidin-biotin-peroxidase complex (ABC ELITE kit, Vector Labs) and developed using the peroxidase method. The sections were mounted on slides, bathed in osmium tetroxide 0.1% for 15-30 s, dehydrated, and covered with coverslips. The individualized immunohistochemical control was processed without the primary antibody, by replacing the primary antibody with normal serum from the animal where the antibody was produced. The slides were analyzed using a light Zeiss Primo Star microscope coupled with a camera. The images were captured and processed using the ZEN 2012 SP1 software (black edition, version 8.1). Table 1 summarizes the different procedures that each group underwent during the experimental period.

**Table 1:** Experimental procedures performed in the different studied groups.

GROUPS	EEG	BUO	GAVAGES	Ur/Cr	IHC
WHI				+	+
WGV			H_2_O	+	+
SGV			Star fruit juice	+	+
NPW		+	H_2_O	+	+
NPS	+	+	Star fruit juice	+	+

### Data analysis

Immunoreactive cells (c-Fos+) were counted in counting areas that could be reproduced on different blades for different groups using the ZEN 2012 SP1 software (black edition, version 8.1). Serum urea and creatinine dosage and count cell values were statistically analyzed using the ANOVA test to identify if there were statistically significant differences between groups at p < 0.01. Differences between groups were analyzed individually using the Student’s t-test with p < 0.01 considered to be significant.

## Results

### Urea and creatinine dosages

The ANOVA analysis showed that the five groups were very different from one another. The non-nephropathic groups (WHI, WGV, and SGV) presented similar urea and creatinine dosages. Urea and creatinine values were respectively three and six times higher in the nephropathic groups (NPW and NPS), which was statistically significant. Data on these dosages are summarized in Table 2 .

**Table 2:** Dosages of creatinine and urea in the different groups expressed as mean ± standard deviation.

	WHI	WGV	SGV	NPW	NPS
Cr	1.62 ± 0.19	1.67 ± 0.09	1.80±0.15	13.02 ± 1.25*	12.90 ±1.12*
Ur	43.73 ± 3.49	43.37± 4.30	42.05± 2.30	139.64±12.00*	154.23±13.30*

*p < 0.01

### Electroencephalogram (EEG)

**Figure 1:**
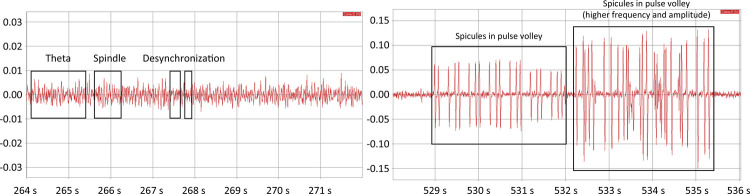
Recordings of electrical activity in the brain of a nephropathic animal that underwent a first gavage with star fruit ( *left* ) and during a seizure attack ( *right* ). On the left, the presence of spindles, as exemplified between 265 and 266, and theta waves, characteristic of relaxed wakefulness, can be seen. Periods characterized by desynchronized waves are also present. During the seizure ( *right* ), a prolonged cluster of spikes with little organization, and increased frequency and amplitudes over time can be seen.

The NPS group showed behavioral signs since the first gavage, including piloerection, discontinued self-cleaning, decreased exploratory activity, drooling, and masticatory automatism, subsequently progressing to myoclonus and generalized tonic-clonic seizures. The visual analysis of the electroencephalograms of the NPS group initially progressed from theta waves ( Fig. 1 , left) to high voltage spikes in weak regular asynchronous pulse volleys between the recorded structures. This disorganization was generalized, and it was not possible to visually identify any propagation between the cortical leads or between the cortical and the hippocampal leads, which suggests that the origin of the epileptic activity was multifocal. The animals were more agitated and would suddenly present with generalized tonic-clonic seizures. These were initially brief; however, after increasing in frequency and amplitude, they became continuous ( Fig. 1 , right).

### Immunohistochemistry

**Figure 2:**
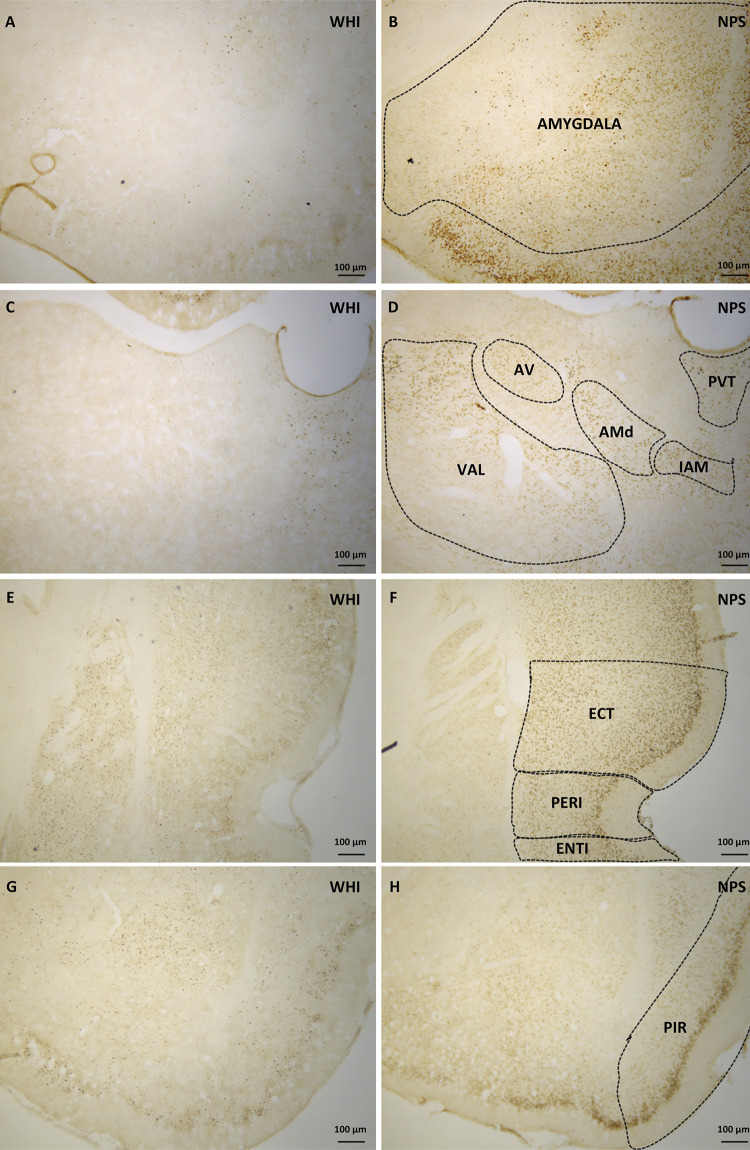
Photomicrograph of rat brain cross-sections that have undergone immunohistochemistry to identify c-Fos protein expression. The figures on the left (A, C, E, G) are from the white groups (WHI). The figures on the right (B, D, F, H) are from the nephropathic star fruit groups (NPS). A/B: Amygdala; C/D: region of the thalamic nuclei, dorsal part of the anteromedial nucleus, anteroventral nucleus, interanteromedial nucleus, paraventricular nucleus, and ventral anterolateral complex (AMd, AV, IAM, PVT, and VAL, respectively); E/F: entorhinal, perirhinal, and entorhinal areas (ENTI, PERI, and ECT, respectively); G/H: piriform area (PIR). There is greater c-Fos expression in the nephropathic groups.

**Figure 3:**
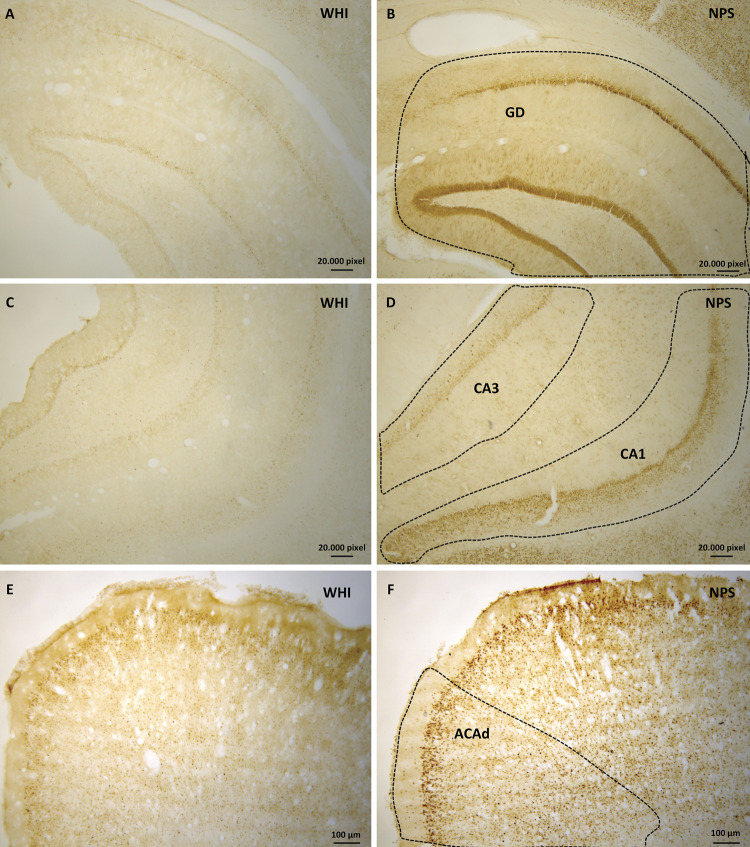
Photomicrograph of cross-sections of the rat brain that have undergone immunohistochemistry to identify c-Fos protein expression. The figures on the left (A, C, E) are from the white groups (WHI). The figures on the right (B, D and F) are from the nephropathic star fruit groups (NPS). A/B: areas of the dentate gyrus (DG); C/D: ventral region of the hippocampus, Ammon’s horn, and CA3 and CA1 fields (CA3 and CA1); E/F: anterior cingulate area, dorsal part (ACAd). There is greater c-Fos expression in the nephropathic groups.

Immunohistochemical analysis showed c-Fos-reactive (c-Fos+) immunoreactive cells in different brain regions and rounded and intensely marked markings were considered positive. The analysis of the entire brain showed immunoreactive areas from the brain stem to the cerebral cortex. Throughout the brain, c-Fos+ cells were found even in animals belonging to the WHI group. Areas that visually presented increased c-Fos expression and belonging to neural circuits potentially involved in convulsive seizures were analyzed. Thus, the following areas were considered for analysis: hippocampus, including the Ammon’s horn, CA1 field (CA1), Ammon’s horn, CA3 field (CA3), and hippocampal dentate gyrus (DG); piriform cortex area (PIR); amygdala complex; entorhinal cortex area (ENTI); ectorhinal cortex area (ECT); perirhinal cortex area (PERI); and the anterior cingulate area, dorsal cortex part (ACAd) and medial anterior thalamus line including the anteromedial nucleus of the thalamus, dorsal part (AMd), anteroventral nucleus of the thalamus (AV), and interanteromedial nucleus of the thalamus (IAM). Figures 2 and 3 show the photomicrographs of the indicated regions.

**Figure 4:**
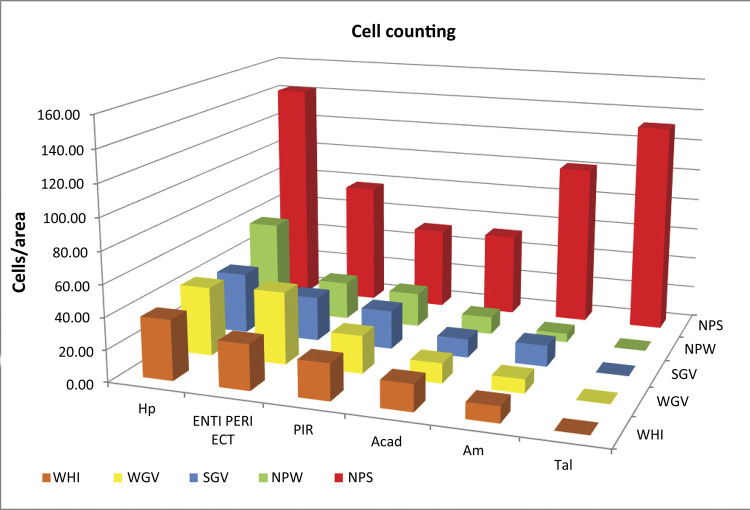
Graph representing the number of cells per counting area. Hp (hippocampus); ENTI, PERI, and ECT (entorhinal, perirhinal, and entorhinal areas, respectively); PIR (piriform area); ACAd (anterior cingulate area, dorsal part); Am (amygdala). Tal (thalamus).

**Figure 5:**
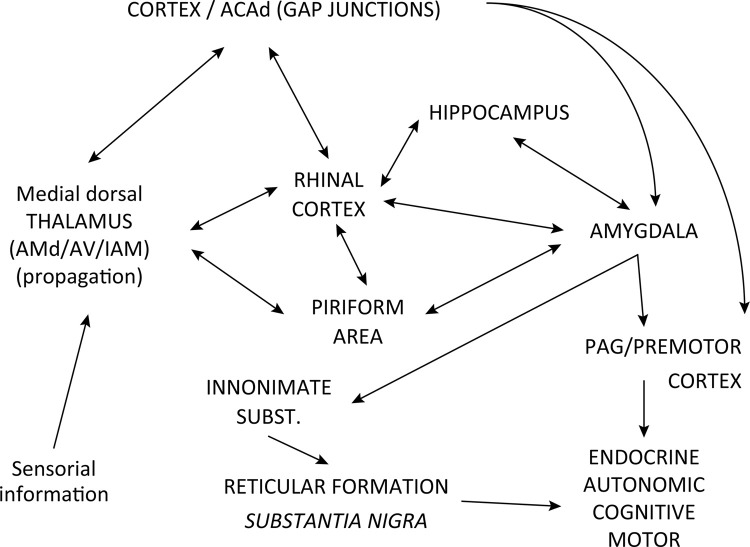
Pathway map summarizing the connections involving c-Fos expression in seizure-related areas of NPS rats. ACAd (anterior cingulate area, dorsal part). AMd, AV, IAM, PVT, and VAL (region of the thalamic nuclei, dorsal part of the anteromedial nucleus, anteroventral nucleus, interanteromedial nucleus, respectively).

Cell counting was used in the statistical analysis. The data can be seen in Table 3 and are expressed as mean ± standard deviation, with statistical differences indicated by a single asterisk. Invariably, in all the nuclei analyzed, the number of c-Fos+ cells in the NPS group was higher than that in the other control groups, including non-nephropathic patients (WHI, WGV and SGV) and the nephropathic control (NPW). In some nuclei/areas, the other control groups showed statistical differences between each other, represented by two asterisks (**). The data indicate that these areas exhibited an increasing state of activity in the NPS group, and that gavage (WGV, SGV, NPW), star fruit administration (SGV), and nephropathy (NPW) were not responsible for the dramatic increase in c-Fos expression, which was due to the association of nephropathy with star fruit administration (NPS). Figure 4 presents illustrative graphs of these data, and the Figure 5 summarizes part of the circuits described and establishes some possible efferent connections related to the onset of a clinical status expressed in seizures.

**Table 3:** Number of c-Fos+ cells/counting area expressed as mean ± standard deviation.

Area	WHI	WGV	SGV	NPW	NPS
Amg	10.06±1.04	8.73±1.03	13.33±1.97**	5.6±1.08	101.6±3.43*
Tal	0 ± 0	0±0	0±0	0±0	132.06±2.72*
ENTI PERI ECT	28.6±4.017	45.6±13.88**	28.06±7.04	24.06±9.92	77.53±8.29*
PIR	23±3.25	23.8±3.45	24.53±2.92	21.73±4.1	51.93±3.2*
Hp	37.8±1.66	43.4±2.15	38.53±2.18	59.4±4.02	142.33±3.37*
ACAd	16.8±10.12**	12.46±1.3	11.73±1.91	11.13±1.68	52.33±2.87*

*NPS statistically different from other groups ( *p<0.001* )

**Control group statistically different from other controls ( *p< 0.001* )

## Discussion

The model previously described by Achar *et al* .^16^ was effective in inducing nephropathy, which was evidenced in the present study by the statistically increased urea and creatinine dosages in nephropathic animals.

Considering that the weight of the animals did not show great variations and that the amount of star fruit juice used (1 mL per gavage) was constant, the volume ingested per unit of weight was very similar across the animals, despite the literature not showing a direct relationship between the amount of star fruit ingestion and the severity of intoxication^4^.

Star fruit intoxication can present different clinical signs, ranging from hiccups to mental confusion, psychomotor agitation, seizures, hypotension, coma, and death. The presence of seizures appears to be a clinical factor of poor prognosis, in which mortality reaches 75%^3,4, 7 - 9^ . Epileptic seizures can be defined as epileptiform cerebral electrical activity caused by sudden, excessive, and hypersynchronous neuronal discharges of focal or generalized origin. When it affects the motor system in general, it is referred to as a seizure. Approximately 8 - 10% of the population may experience seizures during their lifetime, but only 2 - 3% of the population will develop epilepsy. Some factors such as sleep deprivation, alcohol, illicit drugs, certain medications, metabolic disorders, and toxin exposure are factors that can trigger seizures^17^ . The experimental model NPS appears to be related with a metabolic disorder (nephropathy) associated with toxin exposure (star fruit). The animals in the NPS group exhibited seizures that were recorded by EEG. Initially, characteristic waking patterns were observed with rapid and asynchronous theta rhythm without spikes or other pathological signs. Theta rhythm is considered abnormal in human adults, but a moderate amount may be acceptable in the temporal lobes^18^ . However, wakeful rodents have a theta rhythm (7 - 9 Hz) without slow waves of large amplitude, and eventual exhibition of spindles, a characteristic pattern of relaxed wakefulness^19^ . During the gavages, grouped theta waves with increased amplitude and an increasing larger number of spicules in pulse volley were demonstrated, showing multifocal epileptiform activity, which when associated with generalized tonic-clonic seizures confirms the effectiveness of this experimental model to mimic the proposed clinical status.

### C-Fos expression in seizure-related areas of NPS rats

The c-Fos protein has been previously used as a marker in seizure models^20^ . The main finding in this study showed that several brain areas with decreased or no immunoreactivity to c-Fos protein in the animals in groups WHI, WGV, SGV, and NPW however showed high expression of this protein in NPS animals. These areas included those in diverse brain regions, from the brainstem to cortical regions. This study focused on telencephalic areas commonly related to seizures.

The structures involved in a seizure attack can be classified into: 1) triggering epileptogenic areas, which can induce the seizure, but are not necessarily the ones that first present with ictal activity; 2) target epileptogenic areas, which are vulnerable to the development of ictal activity, whether by afferents or by intrinsic circuits, and are usually the first areas to present epileptiform activity; 3) pathways involved in the propagation of the seizure, which are structures that connect triggering areas to target areas, making connections between themselves or between the hemispheres; and 4) modulatory afferents of the triggering or target regions that regulate the seizure threshold; however, unlike the triggering areas, they generally cannot induce a seizure^21^ .

The hippocampus, together with the piriform, amygdala, and entorhinal cortex, are often the first limbic regions that exhibit epileptic discharge, apparently due to their low epileptogenic threshold^21^ . This study corroborates this finding, showing that in hippocampal areas (CA1, CA3, and DG), piriform area, amygdala nuclei, and in entorhinal, perirhinal, and ectorhinal areas, c-Fos expression was statistically higher in the star fruit nephropathic group than in all other groups.

The piriform cortex and peri-amygdaloid area in the temporal lobe constitute the primary olfactory cortex. This region receives afferents from the lateral olfactory stria and has reciprocal connections with adjacent areas, including amygdala, hippocampus, and rhinal cortex, thereby being an integral part of the limbic system. The rhinal cortex is considered a secondary association olfactory area^21^ . This explains, for example, the fact that an odor triggers an emotion, an autobiographical memory, or even a convulsive seizure. The rhinal cortex and its subdivisions are bilateral communication relays between cortex and hippocampus, forming a perfect reverberation model. Functionally, this architecture plays an essential role in declarative memory. However, in pathological conditions, such as seizures, this feedback loop tends to receive excessive stimulation that is characteristic of temporal lobe epilepsy^21 - 23^ .

The piriform and rhinal cortices are sources of excitatory input to the medial dorsal thalamus and hippocampal formation, and are therefore potential seizure-triggering regions as well as propagation zones for seizures originating in the amygdala^22 , 24^ . All these regions were active is the NPS group in this experiment. In limbic convulsions, motor efferences are possibly related to the substantia innominate (ventral pallidum), which receives afferents from the cortex and amygdala, and sends descending projections to reticular formation and substantia nigra among other regions^21 , 24 - 26^ . Therapeutic approaches to epilepsy with a focus on the piriform cortex have been the subject of many studies^23 , 27^ .

This study showed a positive result in the dorsal part of the anterior cingulate area (ACAd). This region is considered an epileptogenic area involved in frontal lobe epilepsy, which is difficult to control. In this area, interneurons are thought to play an important role in synchronizing electrical activity, and the presence of gap junctions allows for the existence of electrical synapses and, consequently, for synchronized action potentials^28^ . Thus, this region is susceptible to the onset of epileptic seizures. However, these seizures commonly present no motor phenotype, and their clinical condition is restricted to cognitive, motivational, and social aspects^28 - 30^ . The c-Fos expression found in the ACAd of the NPS group may be the result of increased activity in other limbic areas with efferent projections to this region. The anterior cingulate cortex can be subdivided into cognitive and affective regions. The affective part establishes, among other functions, connections with the PAG and amygdala, as well as endocrine and autonomic control. The cognitive part is interconnected with the parietal and prefrontal cortex, and with supplementary motor area^29^ .

The amygdaloid complex is in the ventral part of the telencephalon, close to the hippocampus and laterally to the optic tract (OT). This complex consists of several subnuclei with different intranuclear and internuclear connections. Thus, each subnucleus may involve different functions^33^ . Some authors have considered cytoarchitectonic, chemoarchitectonic, and hodological aspects and have proposed the existence of amygdaloid complex subdivisions^31 - 34^ . In this study, different amygdala nuclei were counted separately (including the basal medial nucleus of the amygdala [BMA], basolateral nucleus of the amygdala [BLA], medial nucleus of the amygdala [MEA], and lateral nucleus of the amygdala [LA]). However, the data characteristics were similar across the nuclei, and the pooled data was therefore presented. The amygdala is associated with a wide variety of functions, including physiological, behavioral, and endocrine responses. Generally, these efferent amygdaloid complex responses are due to the processing of emotional stimuli such as fear, anxiety, reward, stress, and the consolidation of aversive memories^33^ . This region also modulates cardiovascular control, influencing heart rate and blood pressure, which is unsurprising, considering the physiological responses to emotions processed by the amygdala^33 , 35 , 36^ . The amygdala, the hypothalamus, and the inferior colliculus are thought to be the integration bridge between seizures originating in forebrain and brainstem circuits^37^ .

The medial dorsal thalamus has bilateral reciprocal connections with several limbic areas, serving as a relay for epileptic discharge to limbic areas^38^ . The thalamus is the largest component of the diencephalon, being part of the limbic circuit, afferent information relay, and relay loop motor programming circuits. Thus, this nuclear complex is one of the most important information relay stations in the brain. Accordingly, in the case of circuits involved with epileptic seizures, the thalamus is a strong candidate for forming pathways involved in the propagation of seizures, connecting triggering to target areas^39 , 40^ . This study reports c-Fos+ expression in the dorsal part of the anteromedial, anteroventral, and interanteromedial nuclei. These nuclei belong to the midline of the anterior thalamic group that is part of the limbic system, and are related to memory and emotional behavior. The nuclei form reciprocal connections with the hypothalamus and cingulate gyrus, in addition to receiving afferents from the hippocampal formation.

## Conclusions

The data obtained in this study shows that nephropathic rats receiving star fruit gavage present increased c-Fos expression in several brain regions involved in convulsive seizures, such as the hippocampus, piriform cortex, amygdaloid complex, entorhinal cortex area, ectorhinal cortex area, perirhinal cortex area, anterior cingulate area, dorsal part of the cortex, and medial anterior line of the thalamus. These areas are often associated with seizures and may be involved either in the genesis and spread or as target areas of epileptogenic conditions.

## References

[6] Chen CL, Fang HC, Chou KJ, Wang JS, Chung HM (2001). Acute oxalate nephropathy after ingestion of star fruit. Am J Kidney Dis.

[7] Carolino RO, Beleboni RO, Pizzo AB, Vecchio FD, Garcia-Cairasco N, Moyses-Neto M, Santos WF, Coutinho -Neto J (2005). Convulsant activity and neurochemical alterations induced by a fraction obtained from fruit Averrhoa carambola (Oxalidaceae: Geraniales). Neurochem Int.

[8] Neto MM, Robl F, Netto JC (1998). Intoxication by star fruit (Averrhoa carambola) in six dialysis patients? (Preliminary report). Nephrol Dial Transplant.

[9] Neto MM, Costa JA, Garcia-Cairasco N, Netto JC, Nakagawa B, Dantas M (2003). Intoxication by star fruit (Averrhoa carambola) in 32 uraemic patients: treatment and outcome. Nephrol Dial Transplant.

[10] Chang JM, Hwang SJ, Kuo HT, Tsai JC, Guh JY, Chen HC, Tsai JH, Lai YH (2000). Fatal outcome after ingestion of star fruit (Averrhoa carambola) in uremic patients. Am J Kidney Dis.

[11] Chang CT, Chen YC, Fang JT, Huang CC (2002). Star fruit (Averrhoa carambola) intoxication: an important cause of consciousness disturbance in patients with renal failure. Ren Fail.

[12] Tse KC, Yip PS, Lam MF, Choy BY, Li FK, Lui SL, Lo WK, Chan TM, Lai KN (2003). Star fruit intoxication in uraemic patients: case series and review of the literature. Intern Med J.

[13] Tsai MH, Chang WN, Lui CC, Chung KJ, Hsu KT, Huang CR, Lu CH, Chuanh YC (2005). Status epilepticus induced by star fruit intoxication in patients with chronic renal disease. Seizure.

[14] Fang HC, Chen CL, Lee PT, Hsu CY, Tseng CJ, Lu PJ, Lai SL, Chung HM, Chou KJ (2007). The role of oxalate in star fruit neurotoxicity of five-sixths nephrectomized rats. Food Chem Toxicol.

[15] Engelborghs S, D’Hooge R, De Deyn PP (2000). Pathophysiology of epilepsy. Acta Neurol Belg.

[16] Fang HC, Lee PT, Lu PJ, Chen CL, Chang TY, Hsu CY, Chung HM, Chou KJ (2008). Mechanisms of star fruit-induced acute renal failure. Food Chem Toxicol.

[17] Garcia-Cairasco N, Moyses-Neto M, Del Vecchio F, Oliveira JA, Santos FL, Castro OW, Arisi GM, Dantas M, Carolino ROG, Coutinho-Neto J, Dagostin ALA, Rodrigues MCA, Leão RM, Quintiliano SAP, Silva LF, Gobbo-Neto L, Lopes NP (2013). Elucidating the neurotoxicity of the star fruit. Angew Chem Int Ed Engl.

[18] Swanson L (2004). Brain maps: structure of the rat brain.

[19] Fonoff ET, Silva CP, Ballester G, Timo-Iaria C (1999). Electro-oscillographic correlation between dorsal raphe nucleus, neocortex and hippocampus during wakefulness before and after serotoninergic inactivation. Braz J Med Biol Res.

[20] Timo-Iaria C, Negrão N, Schmidek WR, Hoshino K, Lobato de Menezes CE, Leme da Rocha T (1970). Phases and states of sleep in the rat. Physiol Behav.

[21] Achar E, Maciel TT, Collares CF, Teixeira VP, Schor N (2009). Amitriptyline attenuates interstitial inflammation and ameliorates the progression of renal fibrosis. Kidney Int.

[22] Gavvala JR, Schuele SU (2016). New-onset seizure in adults and adolescents: a review. JAMA.

[23] Herrmann CS, Strüber D, Helfrich RF, Engel AK (2016). EEG oscillations: from correlation to causality. Int J Psychophysiol.

[24] Vyazovskiy VV, Cirelli C, Tononi G (2011). Electrophysiological correlates of sleep homeostasis in freely behaving rats. Prog Brain Res.

[25] Eells JB, Clough RW, Browning RA, Jobe PC (2004). Comparative fos immunoreactivity in the brain after forebrain, brainstem, or combined seizures induced by electroshock, pentylenetetrazol, focally induced and audiogenic seizures in rats. Neuroscience.

[26] Vismer MS, Forcelli PA, Skopin MD, Gale K, Koubeissi MZ (2015). The piriform, perirhinal, and entorhinal cortex in seizure generation. Front Neural Circuits.

[27] Young JC, Nasser HM, Casillas-Espinosa PM, O’Brien TJ, Jackson GD, Paolini AG (2019). Multiunit cluster firing patterns of piriform cortex and mediodorsal thalamus in absence epilepsy. Epilepsy Behav.

[28] Young JC, Vaughan DN, Paolini AG, Jackson GD (2018). Electrical stimulation of the piriform cortex for the treatment of epilepsy: a review of the supporting evidence. Epilepsy Behav.

[29] Young JC, Paolini AG, Pedersen M, Jackson GD (2019). Genetic absence epilepsy: effective connectivity from piriform cortex to mediodorsal thalamus. Epilepsy Behav.

[30] Maggio R, Lanaud P, Grayson DR, Gale K (1993). Expression of c-fos mRNA following seizures evoked from an epileptogenic site in the deep prepiriform cortex: regional distribution in brain as shown by in situ hybridization. Exp Neurol.

[31] Gale K, Maggio R, Halonen T, Lanaud P, Grayson D (1992). Regional changes in immediate early gene expression following focally-evoked limbic motor seizures. Epilepsy Res Suppl.

[32] Cheng H, Wang Y, Chen J, Chen Z (2020). The piriform cortex in epilepsy: what we learn from the kindling model. Exp Neurol.

[33] Chang WP, Wu JJ, Shyu BC (2013). Thalamic modulation of cingulate seizure activity via the regulation of gap junctions in mice thalamocingulate slice. PLoS One.

[34] Chang WP, Shyu BC (2014). Anterior Cingulate epilepsy: mechanisms and modulation. Front Integr Neurosci.

[35] Chang WP, Wu JS, Lee CM, Vogt BA, Shyu BC (2011). Spatiotemporal organization and thalamic modulation of seizures in the mouse medial thalamic-anterior cingulate slice. Epilepsia.

[36] Swanson LW, Petrovich GD (1998). What is the amygdala?. Trends Neurosci.

[37] McDonald AJ (1998). Cortical pathways to the mammalian amygdala. Prog Neurobiol.

[38] Rasia-Filho AA, Londero RG, Achaval M (2000). Functional activities of the amygdala: an overview. J Psychiatry Neurosci.

[39] Knapska E, Radwanska K, Werka T, Kaczmarek L (2007). Functional internal complexity of amygdala: focus on gene activity mapping after behavioral training and drugs of abuse. Physiol Rev.

[40] Knuepfer MM, Eismann A, Schütze I, Stumpf H, Stock G (1995). Responses of single neurons in amygdala to interoceptive and exteroceptive stimuli in conscious cats. Am J Physiol.

[41] Chiou RJ, Kuo CC, Liang KC, Yen CT (2009). State-dependent amygdala stimulation-induced cardiovascular effects in rats. Chin J Physiol.

[42] Gale K (1992). Subcortical structures and pathways involved in convulsive seizure generation. J Clin Neurophysiol.

[43] Aracri P, Curtis M, Forcaia G, Uva L (2018). Enhanced thalamo-hippocampal synchronization during focal limbic seizures. Epilepsia.

[44] Kim SH, Lim SC, Yang DW, Cho JH, Son BC, Kim J, Hong SB, Shon YM (2017). Thalamo-cortical network underlying deep brain stimulation of centromedian thalamic nuclei in intractable epilepsy: a multimodal imaging analysis. Neuropsychiatr Dis Treat.

[45] Evangelista E, Bénar C, Bonini F, Carron R, Colombet B, Régis J, Bartolomei F (2015). Does the thalamo-cortical synchrony play a role in seizure termination?. Front Neurol.

